# Assessing the Efficacy of First-Aid Measures in *Physalia* sp. Envenomation, Using Solution- and Blood Agarose-Based Models

**DOI:** 10.3390/toxins9050149

**Published:** 2017-04-26

**Authors:** Christie L. Wilcox, Jasmine L. Headlam, Thomas K. Doyle, Angel A. Yanagihara

**Affiliations:** 1 Department of Tropical Medicine, Medical Microbiology and Pharmacology, John A. Burns School of Medicine, University of Hawaii at Manoa, Honolulu, HI 96813, USA; ayanagih@hawaii.edu; 2 Discipline of Zoology, School of Natural Sciences, Ryan Institute, National University of Ireland Galway, Galway H91 W5P7, Ireland; J.HEADLAM1@nuigalway.ie (J.L.H.); tom.doyle@nuigalway.ie (T.K.D.); 3 Békésy Laboratory of Neurobiology, Pacific Biosciences Research Center, University of Hawaii at Manoa, Honolulu, HI 96822, USA

**Keywords:** marine envenomation, Portuguese man o’ war, bluebottle, cnidaria, hydrozoa, first aid, jellyfish, sting

## Abstract

Stings from the hydrozoan species in the genus *Physalia* cause intense, immediate skin pain and elicit serious systemic effects. There has been much scientific debate about the most appropriate first aid for these stings, particularly with regard to whether vinegar use is appropriate (most current recommendations recommend against vinegar). We found that only a small percentage (≤1.0%) of tentacle cnidae discharge during a sting event using an ex vivo tissue model which elicits spontaneous stinging from live cnidarian tentacles. We then tested a variety of rinse solutions on both Atlantic and Pacific *Physalia* species to determine if they elicit cnidae discharge, further investigating any that did not cause immediate significant discharge to determine if they are able to inhibit cnidae discharge in response to chemical and physical stimuli. We found commercially available vinegars, as well as the recently developed Sting No More^®^ Spray, were the most effective rinse solutions, as they irreversibly inhibited cnidae discharge. However, even slight dilution of vinegar reduced its protective effects. Alcohols and folk remedies, such as urine, baking soda and shaving cream, caused varying amounts of immediate cnidae discharge and failed to inhibit further discharge, and thus likely worsen stings.

## 1. Introduction

Cnidarian envenomation represents a significant medical burden worldwide. Envenomation involves the triggering and discharge of hundreds to thousands of specialized cells, called cnidocytes or nematocytes. Certain cnidocytes contain penetrant cnidae; these microscopic venom-injecting capsules are called nematocysts. Upon contact, nematocysts can deliver a potently toxic chemical cocktail into unsuspecting prey or human victims [1]. Pelagic cnidarian species that possess long tentacles armed with cnidocytes are colloquially known as “jellies” or “jellyfish”, and represent the most dangerous members of the phylum. Jellyfish envenomation can range from mild to life threatening, depending on the species involved, and the amount of venom delivered [2,3,4].

The marine siphonophores *Physalia utriculus* (Indo-Pacific; commonly called bluebottle) and *P. physalis* (Atlantic; referred to as Portuguese man o’ war) are dangerous marine organisms commonly found in tropical oceans. Each consists of an above-water float (pneumatophore), various specialized feeding and reproductive structures and long, stinging tentacles used for capturing prey. These marine stingers are often found in large groups or “armadas” that can be blown onshore by strong winds. While deaths from *Physalia* stings are rare [5,6], stings can be excruciating and lead to systemic complications, including headache, vomiting, abdominal pain and diarrhea [1,2,3,4,7,8,9]. Their penetrant cnidae (nematocysts) have long tubules, which can deliver venom nearly 1 mm into tissues and remain embedded, likely causing secondary immunological reactions [10]. A recent study has described a marked increase in *Physalia* stings along the French Atlantic coasts [11], further stressing the urgency of standardized, evidence-based care for *Physalia* stings.

While vinegar dousing has been recommended historically [12], there is no universally accepted first aid for *Physalia* stings, and first-aid approaches have recently become increasingly controversial [13]. Because studies have shown that physiological responses to all cnidarian envenomation are dose-dependent, and only a small portion of available cnidae discharge upon initial contact [14], one of the key first steps in first aid is to ensure the safe removal of adherent tentacles and undischarged cnidae (which are capable of firing for at least two weeks after their separation from the tentacle [15]). Thus, a critical initial first-aid goal is to remove stinging material without increasing the amount of venom injected or the number of cnidae discharged into the skin, and thus rinse solutions that irreversibly inhibit cnidae discharge are preferred. Rinses that trigger functional cnidae discharge to effect further impalement of the epidermis with venom deposition cause more harm than good, potentially turning a mild or moderate sting into a severe one. In a somewhat counter-intuitive manner, however, certain rinses such as sea water which may appear at first consideration to be “inert” and represent a “do no harm” type of approach, simply allow the tentacles, as well as dissociated intact cnidae, to be moved further on the surface of the skin at which point the tentacle cnidae or isolated intact cnidae sting, thus “spreading” the sting area and increasing the “venom load”. Further, another potential area of unexpected contradiction is the finding that while some rinses induce discharge of cnidae along an isolated tentacle on a microscope slide, the discharged cnidae are not functionally capable of impaling skin and/or the venom is immediately inactivated. These two findings caused us to carefully re-examine the extant literature. The prevailing premise has been that microscope slide assays of tentacle responses to potential rinse solutions directly correlate to venom load and thus utility in sting management. The finding that this is not the case [16] is pivotal in efforts to develop optimal sting management protocols.

The few investigations on potential rinse solutions for *Physalia* stings have been fraught with inconsistent and contradictory results. Initial studies reported that alcohols cause massive cnidae discharge, while discharge is absent or inhibited in the presence of weak acetic acid solutions (~5%, in distilled water), or household vinegar [17], a finding confirmed in another hydromedusan species, *Olindias sambaquiensis* [18]. Some reduction in pain was also noted with vinegar application on *Physalia* stings in a prospective controlled clinical trial (*N* = 20) [19]. However, microscopic examination in another study [20] found moderate cnidae discharge in the presence of vinegar (an average of “2” out of “5”, with 5 being “maximal” discharge), and a more recent paper [21] observed what they described as a relatively high degree of discharge among the data observed (nematocysts per 1 mm of linear tentacle; roughly double the discharge observed after exposure to 1:10 dilution of 70% ethanol) from *Physalia physalis* tentacles with the application of dilute (1:10) 5% acetic acid. Because of the variability in laboratory-based results, it was suggested in the 1980s that vinegar use for *Physalia* stings be discontinued [20,22]. This suggestion has become standard; medical doctors warn of vinegar’s danger, even calling its use “forbidden” in the case of *Physalia* stings [23] (p. 86), and national and international organizations often question or warn against the use of vinegar if *Physalia* is implicated (e.g., [24,25,26]). The recommendation has stuck despite the fact that the combined evidence from the highest-quality studies supports vinegar as the best course of first aid [27].

In this study, we sought to investigate the degree of cnidae discharge during a sting event. We then sought to re-examine potential rinse solutions previously tested by other investigators, as well as popular folk remedies (such as urine), to rigorously determine which solutions inhibit *Physalia* cnidae discharge to determine the best choices for first-aid use. We compared the results for Atlantic and Pacific *Physalia* species to look for species-specific responses, as well as determine whether there is likely to be a universally applicable solution for *Physalia* species. Lastly, we examined whether the results of solution-based methods that evaluate cnidae discharge correlate to functional measures of venom load (in a blood agarose-based model), to further evaluate whether solution-based studies are clinically relevant.

## 2. Results

### 2.1. Envenomation Model and Cnidae Discharge

**Figure 1:**
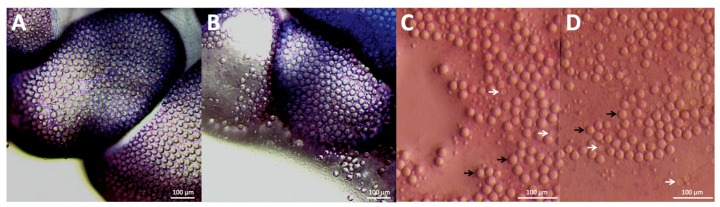
Estimation of cnidae discharge during a sting event. Cnidae visualized (**A**) before and (**B**) after stinging in the blood agarose model (*N* = 4); an estimated 5.2 ± 1.5% of the tentacle’s cnidae sloughed off and remained adherent to the agarose, which are shown in (**C**,**D**). Panel (**C**) shows adherent cnidae after the 10-minute sting, while (**D**) shows the increased discharge of adherent cnidae after the application of site pressure. In both, examples of undischarged cnidae are indicated with black arrows, while discharged cnidae are indicated with white arrows. Site pressure led to about three times as much discharge from adherent cnidae.

**Figure A1:**
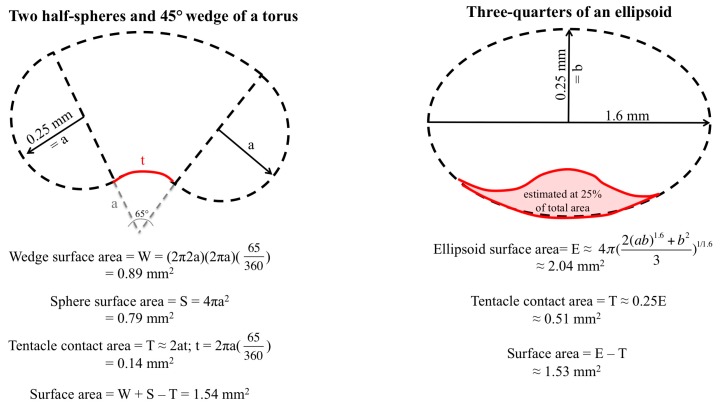
Two methods for estimation of dactylozooid bead surface area for calculation of percent cnidae discharge during a sting event. As both methods produced similar results, 1.54 mm^2^ was used as the bead surface area.

We previously reported the development of a spontaneous sting model [14], in which live tentacles sting agarose containing suspended live red blood cells; the rate of cnidae discharge per unit tentacle length in these modeled stings is indistinguishable from actual human envenomations. We used the same methods to model *P. utriculus* stings and estimate the number and percent of total large and small penetrant cnidae or nematocysts (specifically, heterotrichous anisorhizas [10]) that discharge per length of stinging tentacle as well as the number of anisorhizas left upon point contact with the blood agarose (all results in this section are presented as mean ± standard error). As a first approximation of the potential maximal number of nematocysts per unit length of stinging tentacle, large anisorhiza packing density on the dactylozooid tentacle was quantified from microscopic examination prior to stinging (868 ± 25 cnidae per mm^2^; see Figure 1). The calculated cnidae density was applied to the surface area of each nematocyst cluster or “bead” (which was estimated using two methods to be ~1.54 mm^2^; see Figure 1A and Figure A1), for a total bead cnidae count of 1337 ± 38. The number of beads per stinging millimeter of tentacle was determined from microscopic images (4 beads per mm, contracted tentacle length, while stinging), to yield a value of 5347 ± 154 cnidae per mm of stinging tentacle.

After a 10-minute sting, 412 ± 34 cnidae per mm of tentacle remained on the agarose, which was 5.2 ± 1.5% of the tentacle’s available cnidae. Most of the adherent cnidae were undischarged; only 11.8 ± 2.8% of cnidae on the agarose were discharged (24 ± 4 per mm of stinging tentacle). Thus, during the sting event, only 0.5 ± 0.1% of tentacle large anisorhizas fired.

When slight pressure (2.5 g per cm^2^) was applied using a weighted microscope slide over the adherent cnidae, the percent discharge roughly tripled from 11.8 ± 2.8% to 32.3 ± 7.2% (Figure 1C,D). Tubules from these additionally discharged cnidae were observed penetrating into the agarose, leading to observable increases in hemolysis.

### 2.2. Testing of Potential Rinse Solutions Using the Tentacle Solution Assay

**Figure 2:**
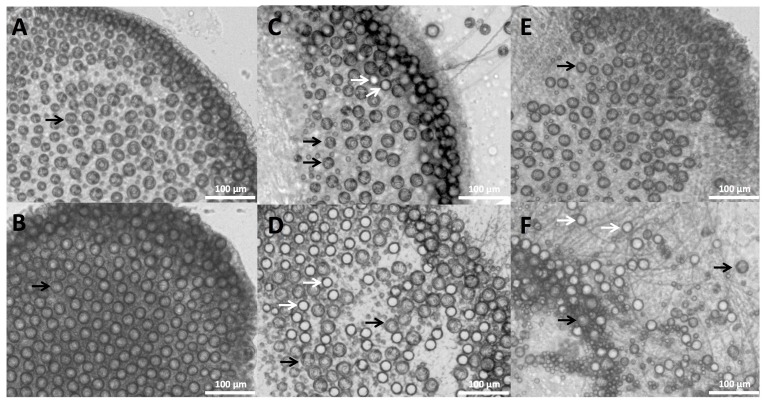
Microscope images of *P. utriculus* tentacles 10 min after exposure to: (**A**) seawater; (**B**) vinegar; (**C**) urine; (**D**) ethanol; (**E**) Sting No More^®^ Spray; or (**F**) 30 s of pressure in the TSA. Discharged cnidae appear empty (lighter than the background or even white), though the extended tubule is not always visible (white arrows), while undischarged cnidae are darker than the background with a coiled tubule visible (black arrows). No discharge was seen in (**A**,**B**,**E**); moderate discharge is visible in (**C**); and maximal discharge is seen in (**D**,**F**).

To compare with previous investigations, we examined the in vitro effects of potential rinse solutions using the Tentacle Solution Assay (TSA). We were able to visualize cnidae in detail and readily calculate percent discharge in response to different test solutions (Figure 2) [14]. Given that these animals live in seawater, we used the response to seawater (0.45 μm filtered) as a baseline; not surprisingly, seawater elicited negligible discharge from both species (Table 1). Ethanol elicited significant discharge in a concentration-dependent manner. Similarly, moderate pressure resulted in marked numbers of cnidae discharge events. Slight differences between the species were found in their responses to freshwater (tap water) and urine (freshly collected), with the *P. utriculus* less responsive than *P. physalis* (Table 1). Three different types of vinegar and Sting No More^®^ Spray elicited no significant discharge in either species (Table 1).

As few differences were noted between the two species, we further examined the ability of solutions to inhibit cnidae discharge in depth using *P. utriculus*. We compared readily available and folk remedies with different vinegar solutions, other acidic solutions, alcohols and the new Sting No More^®^ technology (Table 2).

Solutions differed significantly in their induction of discharge (one-way ANOVA, *p* < 0.001). Several readily available solutions frequently recommended, including urine, shaving cream, baking soda, and lemon juice, induced significant discharge (Table 2 and Table A2). Differences were also found in our inhibition tests (two-way ANOVA, *p* < 0.001). While vinegar irreversibly inactivated cnidae, most solutions, including vinegar dilutions, did not completely inhibit discharge. Some solutions appeared to partially reduce discharge, as they had significantly less pressure-induced discharge than seawater but more than seawater alone (Sidak post-hoc comparison of solution + pressure to seawater + pressure *p* < 0.05; Table 2 and Table A2). However, both urine and lemon juice caused significant discharge initially, and thus have the potential to increase the number of penetrant stinging cnidae even without pressure. All vinegars tested as well as 5% acetic acid and Sting No More^®^ Spray prevented pressure-induced discharge (Sidak post-hoc comparison to solution-only *p* > 0.05).

### 2.3. Evlauation of Potential Rinse Solutions Using the Tentacle Blood Agarose Assay

To further examine solutions with promise based upon the TSA results and authenticate or “ground-truth” the use of the TSA in evaluating first-aid solutions, functional activity assays were conducted using live tentacles in ex vivo Tentacle Blood Agarose Assays (TBAA) to evaluate whether a subset of the rinse solutions tested in the TSA as well as post sting topical hot-, ambient- or cold-pack exposure led to increases or decreases in hemolytic zone formation. Live red blood cell hemolysis is a direct venom activity assay and a functional metric of various venom constituents including venom cytolysins, proteases, and lipases.

**Figure 3:**
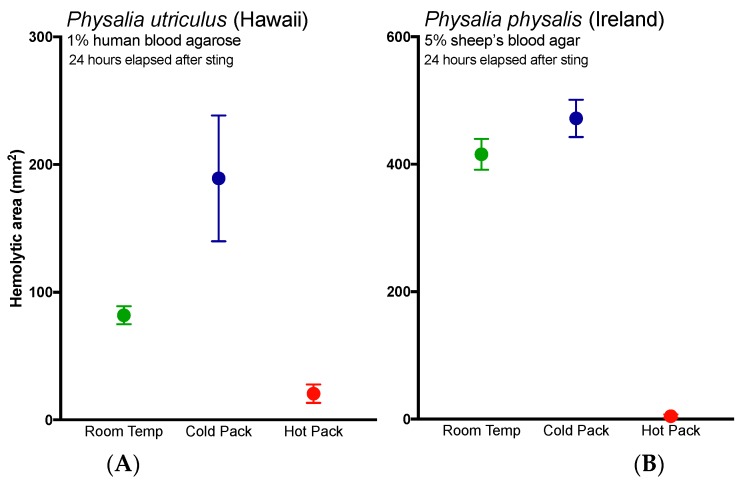
Size of venom-induced hemolytic zone over time using the TBAA model when stings were treated with hot packs, cold packs, or kept at room temperature for: (**A**) *P. utriculus* stings of human blood agarose or (**B**) *P. physalis* stings of sheep’s blood agar plates. Significant differences between the treatments were found for both (Kruskal–Wallis Test, *p* < 0.0001 for *P. physalis* and one-way ANOVA, *p* = 0.0097 for *P. utriculus*). Cold packs resulted in significantly larger hemolytic zones after 24 h when compared to hot packs (post-hoc Fisher’s LSD; *p* = 0.0034 and *p* < 0.0001 for (**A**,**B**), respectively).

Temperature treatments had significant impacts on the size of the hemolytic zone 24 h after the sting, a time dependent indicator of both venom amount and activity (Kruskal–Wallis Test, *p* < 0.0001 for *P. physalis* and one-way ANOVA, *p* = 0.0097 for *P. utriculus*; Figure 3). While the application of cold packs has been considered a “do no harm” first aid, a marked increase in hemolytic zone was observed, which continued to enlarge over time. Specifically, after 24 h, *P. utruculus* stings treated initially with instant cold packs for 20 min, similar to those commonly carried by emergency personnel, were an average of 2.3 times worse than stings left at room temperature and 9.2 times worse than those treated with reusable instant hot packs, differences which were significant (post-hoc Fisher’s LSD; *p* = 0.0290 and *p* = 0.0034, respectively), while *P. physalis* stings treated with ice packs for 40 min were 13% worse than those left at room temperature and more than 100-fold worse than those treated with hot packs for the same time (Dunn’s multiple comparisons, *p* = 0.3964 and *p* < 0.0001, respectively).

**Figure 4:**
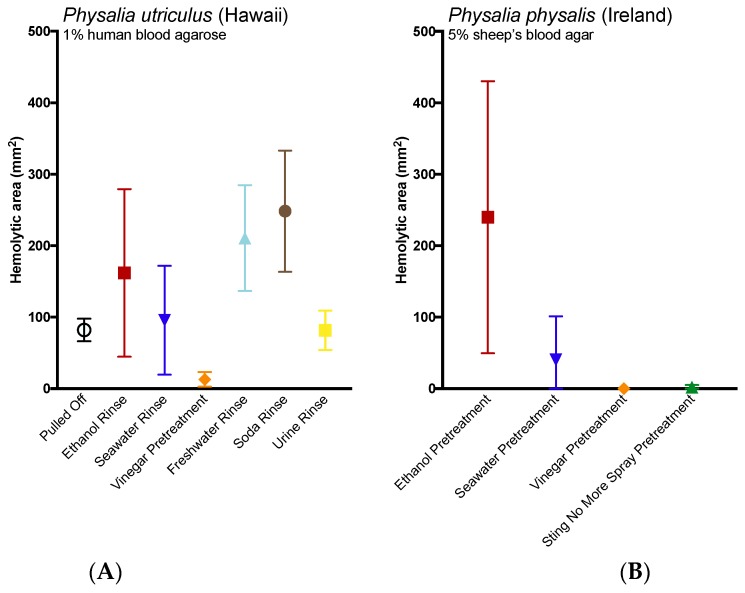
Size of venom-induced hemolytic zone over time using the TBAA model: (**A**) when *P. utriculus* tentacles were doused halfway through the sting with seawater (dark blue), soda (brown), ethanol (red), freshwater (light blue), or urine (yellow), or were pretreated with vinegar for 30 s (orange) using the human blood agarose, or (**B**) when *P. physalis* tentacles were pretreated with seawater (dark blue), ethanol (red), vinegar (orange) or Sting No More^®^ Spray (green) and tested using sheep’s blood agar. Significant differences between the treatments were found for both (Kruskal–Wallis Test, *p* = 0.0162 for *P. physalis* and one-way ANOVA, *p* < 0.0001 for *P. utriculus*). The addition of soda, freshwater and ethanol increased the size of the hemolytic area of *P. utriculus* stings after 24 h (*p* = 0.0007, = 0.0065, and = 0.0791 respectively compared to the no solution control in Fisher’s LSD post-hoc analyses), while pretreatment with vinegar led to a smaller area of lysis (Fisher’s LSD, *p* = 0.1259). Pretreatment with vinegar also reduced the lysis for *P. physalis,* particularly when compared with pretreatment with ethanol (*p* = 0.0285 in Dunn’s multiple comparison test).

The application of potential rinse solutions also had a significant impact on hemolytic zone size for both species (Kruskal–Wallis Test, *p* = 0.0162 for *P. physalis* and one-way ANOVA, *p* < 0.0001 for *P. utriculus*; Figure 4). Three solutions—soda, freshwater and ethanol—increased the size of the hemolytic area from *P. utriculus* tentacles after 24 h when compared to tentacles removed by pulling (a no solution control; 3.0, 2.6 and 2.0 times larger hemolytic zones; Fisher’s LSD, *p* = 0. 0007, *p* = 0.0065, and *p* = 0.079, respectively). Vinegar pretreatment reduced the hemolytic zone area (6.4 times smaller at 22 h; *p* = 0.1259). Similarly, vinegar and Sting No More^®^ Spray pretreatment reduced the hemolytic zone area of *P. physalis* tentacles, although the results were not significant.

## 3. Discussion

The integrated structural and functional approach taken in this study demonstrates a potential explanation for the level of controversy in the literature related to sting first aid management. Based upon our findings in cubozoa [15] and other species including the current study of the siphonophore *Physalia*, it appears that TSA-based physical data and the presence or absence of discharged cnidae, is not a useful predictor of sting severity, i.e., a functional metric of venom induced tissue damage. In summary, this study further supports the use of cnidae-inactivating rinse solutions as a primary step in the cnidarian envenomation first aid. We found that roughly 0.5% of tentacle cnidae discharged within the first 10 min of tentacle contact to the blood agar blood cell tissue model; in previous work, stinging of this proxy was indistinguishable from an authentic human sting [14]. A maximum of about 50% discharge was elicited when tentacles were stimulated chemically or mechanically (Table 1 and Table 2), indicating that there may be a biological constraint on the total amount of cnidae available to discharge. Given that cnidae are spent and replenished during feeding, this observation makes sense, as its very likely many cnidae may be immature. If we assume that our maximal inducible discharge represents the maximum available cnidae during a sting, then we would estimate that about 1% of available cnidae discharge during the initial sting event. Many of the undischarged yet available cnidae could be shed during tentacle contact, as we found large numbers of undischarged cnidae on the agarose surface. Many of these cnidae were able to fire when gently pressed, and thus should be considered active and dangerous. Between the tentacle itself and the contact area, nearly 100 times the number of stinging cnidae may be available to discharge into an unfortunate sting victim, and thus safe and effective removal of adherent tentacles and undischarged cnidae should be considered a first-aid priority.

Overall, we found strong similarities in the responses of both species to various solutions. In two cases, however, we saw slight differences in vitro: the Pacific *P. utriculus* was less responsive to both urine and tap water than the Atlantic *P. physalis*. These discrepancies may be due to differences in collection method rather than representing species-specific responses. All Pacific *Physalia* used in the TSAs were collected after they washed ashore and were beached for some time (<2 h beached, but exactly how long for each individual was unknown). The Atlantic specimens used for the TSAs, on the other hand, were collected live from the Gulf Stream using nets. As such, they would not have experienced any period of time out of water or dehydration or depletion due to rolling contact with sand and may thus have been more sensitive to certain solutions, especially aqueous ones.

To our knowledge, this study represents the first laboratory test of shaving cream on *Physalia* tentacles, which is often suggested by authoritative web sources (e.g., [28,29]) including the United Kingdom National Health Service [25] to be used in combination with a razor or credit card to “shave” away adherent cnidae. As scraping the skin would undoubtedly act to apply pressure to any adherent cnidae and therefore increase discharge [15] (in our test, slight site pressure increased adherent discharge by three fold; see Figure 1C,D), we tested the ability of shaving cream and other compounds to prevent cnidae discharge regardless of whether they induce discharge. No readily available solutions outside of vinegars fully inhibited pressure-induced discharge, and many showed large increases in discharge after pressure application (Table 2), thus our data would suggest scraping the site should not occur unless the area is doused with vinegar first.

These results strongly supported the use of vinegar as a rinse solution; vinegars acted as potent inhibitors of both chemically-induced and pressure-induced cnidae discharge (Table 1 and Table 2). We found that several varieties of vinegar as well as laboratory-made dilute acetic acid solutions inhibited cnidae discharge in both species no matter the stimulus (*P. physalis* results can be found in Table A1). Further, the pre-treatment of tentacles for 30 s with vinegar significantly reduced their ability to sting (Figure 3). These results are concurrent with Burnett et al., 1983 [17], but contrast three other studies [20,21,22]. However, a closer examination of the methods used in the contrasting studies may explain the apparent discrepancies in results. Exton [20] rated discharge from vinegar, methylated spirits and water relative to the “maximal” (a score of 5) and “minimal” (a score of 0) discharge seen; vinegar scored 2/5, spirits scored 5/5, and water scored 0/5. It is difficult to compare these results with our own as the scale is unclear and there was no indication of variability or error; at best, we can say that in their study vinegar was substantially better than ethanol, which would be consistent with our results. Birsa et al. [21] found that “5% acetic acid” induced discharge in *P. physalis*; the addition of acetic acid led to nearly double the amount of discharge of cnidae as “70% ethanol” (100 and 53 ± 26 nematocysts per mm of tentacle, respectively). Their alcohol results contrast Exton’s as well as the results presented here, as we found 70% ethanol induced dramatically more discharge in both species tested (Table 1). However, Birsa et al. [20] noted that their discharge metric—the number of nematocysts discharged per millimeter of tentacle length—was flawed due to the variability of nematocyst density on tentacles, and thus they ”suggest that at best it is a semi-quantitative” (p. 427). It is unclear why they did not count undischarged cnidae to obtain a standardized value, but since there is no indication of how variable the cnidae density was between tentacles or what the average density was, it is unclear whether the count for 5% acetic acid is comparable to the other counts in their table, or what degree of discharge their counts represent (if our tentacle counts of cnidae density were used, then 100 cnidae per mm would translate to 1.8% discharge, as compared with our findings of 0.5% for 5% acetic acid and 1.7% for 0.5% acetic acid). However, perhaps most confounding is that Birsa et al. [20] also reported in their methods that they added 100 µL of their test solution to a well containing 1 mL of seawater and the tentacle, which would have resulted in a final concentration of 0.5% acetic acid rather than 5% and 7% ethanol rather than 70%. Further complicating comparisons is that acetic acid appears to have been only counted once (the value for acetic acid does not have error margins while values for their other solutions do), thus it is impossible to determine if that count of discharged cnidae is representative of the animals’ response. We found in our studies that the dilution of acetic acid solutions completely abolished their protective effects, and that diluted solutions could even induce slight discharge (Table 2); meanwhile, dilute alcohol solutions induced markedly lower discharge than concentrated alcohols (Table 1). Based on microscope images alone, it appears that very little discharge was induced in *P. physalis* overall, as only a few isolated tubules can be seen (p. 428, Figure 1A for acetic acid, Figure 1D for ethanol)—which would be consistent with the results presented in Table 2 for diluted solutions. The only other paper to report discharge from vinegar in *Physalia* had methods too vague to compare [22]. Fenner et al. stated that in their experiments, discharge occurred in “up to 80% of nematocysts”, but that this only occurred in “some, not all of the specimens” and that “conflicting results were obtained if other parts of the tentacles were tested, even from the same jellyfish”, and that “approximately 30%...showed some discharge” [22] (p. 499). Studies on other hydrozoans have found limited or no discharge induced by vinegar and strong discharge from alcohols, similar to our results [18,30].

The inhibition of discharge by vinegar was not due to its acidic nature alone, as other solutions with similar pH were unable to prevent discharge. Vinegar and dilute acetic acid solutions have long been used to preserve food and fix tissues [31]. Vinegar or ~5% acetic acid causes marked swelling of collagen by increasing the absorption of water [31]. It has long been documented that vinegar exposure “fixes” nematocysts rendering them incapable of functional discharge. This is likely due to the fact that nematocyst capsule walls are comprised of collagen and that acetic acid-induced swelling irreversibly alters the structural features required for functional firing [10]. We found strong evidence against the use of ethanol and freshwater, as these solutions significantly increased cnidae discharge (Table 2) and worsened stings in the TBAA model (Figure 3). In addition, our results do not support the use of the most infamous sting treatment: urine. It is unclear exactly when the use of urine for jellyfish stings became popular (certainly, a scene featuring the treatment method in the sitcom “Friends” aided its spread [32]), but it has become one of the most persistent myths in toxinology. Urine induced significant discharge in both species of *Physalia* tested, with discharge on par with ethanol application for *P. physalis*, the larger and more dangerous species. While urine did not cause significant increases in hemolysis in our functional assay, we did not have the opportunity to test *P. physalis* using the TBAA. Furthermore, even with the less-responsive species, we did detect an increase in the hemolytic zone size after 22 h, which was not statistically significant perhaps due to the small sample sizes (*N* = 5). Further investigations into the effects of urine in both solution-based and functional assays should be conducted, and, in the meantime, its use for treating stings discouraged.

Although we did not see a significant increase in discharge from the application of soda in the TSA (Table 2), it most strongly increased hemolysis in the TBAA (Figure 4). Why soda caused such damage in the ex vivo model is unclear; soda alone did not cause hemolysis. It is possible that sugars or other compounds present or carbonation acted to enhance cnidae discharge in the presence of perceived prey (blood cells). Further tests are required to examine the basis of the increase in hemolysis triggered by the addition of soda. However, perhaps more importantly, this discrepancy highlights the need for investigating potential first aid approaches using multiple methods, as a lack of induction of discharge in the TSA does not consistently correlate with the functional metric of venom activity, hemolysis, and thus is of questionable value as a determinant of efficacy of first aids. Live tentacle cnidocytes are known to be associated with sensory hair cells [33], and cnidae triggering involves both chemo- and mechano-sensory input. From a methodological standpoint, the effects of solutions on a tentacle laying on an inert glass surface differ from tentacle contact to the epidermis of live prey. Thus, the TSA does not necessarily predict potential changes in cnidae triggering in the presence of human cells, nor can it account for the physical movement of the tentacle or cnidae that may be induced by the application of a first-aid. For example, rinsing with an “inert” solution (defined as not triggering discharge in the TSA) may have deleterious effects in functional models, as the act of rinsing could cause tentacles to shift or roll. Such movements could place more cnidae in contact with the skin locally, increasing stinging, as well as move the stinging cells to previously untouched patches of skin, increasing the size of the sting site. Experimentally, this has been demonstrated to occur with cubozoans; seawater rinsing was found to worsen stings even though it does not directly induce discharge [15]. While the TSA can be used as a preliminary screen to look for potent cnidae triggers, which can be dangerous to apply to stings (such as ethanol), or effective inhibitors of discharge (such as vinegar), it should not be considered a conclusive test for efficacy and safety, especially for solutions which neither trigger nor inhibit cnidae discharge in vitro.

Lastly, these results bolster previous studies that support the use of heat in the treatment of *Physalia* stings [34,35], as the application of heat significantly reduced hemolysis in the TBAA (Figure 3A, red line versus black line). The application of cold not only failed to reduce hemolysis, it worsened stings (Figure 3, blue lines). While it is possible that the application of cold increased discharge of shed cnidae, thus directly increasing venom load, we posit an alternative possibility. These results suggest that while the physical trauma of a sting and initial pain are acute events, venom pathogenic mechanisms may have a protracted kinetic course. The enzyme kinetics have yet to be carefully elucidated but time course studies (data not shown) reveal that the lipase reaction kinetics exhibit substrate to product conversion over 12 h comparable to other cnidarian lipases [36]. The effects of initial temperature treatments on such kinetics are not known; however, it is reasonable to believe that cold treatment could enhance venom activity, as previous studies have found that activity is preserved at cold temperatures and abolished by hot ones (for a review, see [37]). Further, anecdotal accounts have noted increases in pain or “reawakening” (rebound) of the sting upon rewarming after the application of cold [38,39]. To our knowledge, this is the first quantitative report of both acute and protracted harm from the use of cold packs, and warrants immediate reevaluation of the use of cold packs in the treatment of cnidarian envenomations. Studies evaluating the possible injurious effects of cold pack application in more lethal species should be conducted expediently.

## 4. Conclusions

It is noteworthy that the number of cnidae discharging in a *Physalia* sting represents a small fraction of the packed cnidae along the tentacle surface (≤1.0% of available cnidae). For this reason, the method of tentacle and shed undischarged cnidae removal or rinsing has the potential to dramatically increase the sting area and thus the sting severity. We found that while various types of vinegar-inhibited discharge in both *Physalia* species, even a slight dilution of vinegar markedly reduced inhibitory effects or led to partial cnidae discharge. Further, a 30-second treatment of vinegar reduced stinging in our blood agarose model. The inhibition of discharge was not simply a function of pH, as other acidic solutions did not have these results. Alcohols strongly induced discharge in a concentration-dependent manner, and resulted in clear evidence of increased hemolysis, and thus should be avoided. Other possible first aids, including urine, baking soda, shaving cream, dish soap, lemon juice, and regular cola, failed to prevent pressure-induced discharge, and most elicited discharge upon contact. This study demonstrates the need to utilize both physical reactive assays such as the assessment of cnidae discharge as well as functional assays of venom activity in order to more fully explore the efficacy of potential first aid approaches. The effects of carbonated soda beverage in particular highlighted the need for multiple avenues of investigation when evaluating first aids, as it was relatively inert in vitro but caused the greatest increase in the area of hemolytic zone. Based on these findings, vinegar use is highly supported as the primary rinse solution for *Physalia* stings, returning to recommendations made over thirty years ago. Since vinegar is already the go-to rinse solution for other cnidarian stings, removing the *Physalia* caveat will simplify treatment recommendations making them easier for the general public and first responders to remember and apply correctly. In addition, the temperature results underscore the need for proper evaluation of first aids prior to the adoption of their use, as cold packs severely exacerbated stings. If cold or ice pack application results in similar increases in venom activity that has been shown with more lethal species [16], then it could be a contributing factor to morbidity and mortality worldwide.

## 5. Materials and Methods

The chemicals for the solutions used in all assays are as follows: seawater (locally collected, 0.45 μm filtered), white vinegar (Bakers and Chefs CJ314, SAM’s West Inc., Bentonville, AR, USA), cider vinegar (Heinz Brand, H.J. Heinz Corp., Pittsburgh, PA, USA), malt vinegar (Heinz Brand, H.J. Heinz Corp., Pittsburgh, PA, USA), balsamic vinegar (Safeway, Pleasanton, CA, USA), shaving cream (Gillette Regular Foam, Procter and Gamble, Boston, MA, USA), baking soda (mixed 3:1 with filtered seawater; Arm and Hammer, Church and Dwight Co., Princeton, NJ, USA), dish soap (Dawn brand, Procter and Gamble, Cincinnati, OH, USA), acetic acid (Fisher Scientific, Fair Lawn, NJ, USA), lidocaine (4% in 150 mM saline; MP Biomedicals LLC, Solon, OH, USA), ethanol (Pharmco-Aaper, Brookfield, CT, USA), isopropanol (Fisher Scientific), copper gluconate (30 mM in 150 mM saline; Strem Chemicals, Newburyport, MA, USA), magnesium sulfate (50 mM in filtered 150 mM saline; Fisher Scientific), sodium chloride (Fisher Scientific), hydrochloric acid (Fisher Scientific), lemon juice (Mott’s LLP, Pano, TX, USA), regular cola (Pepsi; Pepsico, Purchase, NY, USA), Sting No More^®^ products (contents include copper gluconate, urea, and magnesium sulfate; Alatalab Solutions™ LLC, Honolulu, HI, USA), copper gluconate (30 mM in 150 mM saline; Strem Chemicals, Newburyport, MA, USA). Urine was freshly collected from a willing volunteer prior to use and 0.45 μm filtered. The vinegar used in Ireland was Tesco distilled vinegar (produced in the UK for Tesco Stores Ltd., Chestnut, UK).

### 5.1. Animal Collection

Live *P. utriculus* were collected after sustained strong winds from Kailua Beach on the eastern side of Oahu (21°23′47.7″ N 157°43′29.2″ W). Animals were placed in individual containers with ample seawater and kept cool until use. Live *P. physalis* for solution-based assays were collected from gulfstream waters off Miami’s South Beach (25°50′48.3″ N 79°48′57.7″ W). Animals were netted and bagged in gallon Ziploc bags with ample seawater and kept cool until use. Animals were warmed to room temperature in 0.45 μm-filtered seawater before their tentacles were used. All experiments were conducted within 72 h of collection. Live *P. physalis* for blood agar assays were also collected from several beaches (Derrynane, Co. Kerry, Youghal, Co. Cork and Ardmore, Co. Waterford, all in Ireland) during periods of strong southwesterly winds. The majority of specimens were collected within a few hours of stranding and several were collected from the incoming tide. The specimens were typically small, with pneumatophore lengths of 60 mm to 150 mm. They were kept in ample amounts of ambient seawater for up to 24 h, before having their fishing tentacles excised. Tentacles were excised close to the pneumatophore using a pair of dissecting scissors and were handled with a fine forceps before being immediately used in the experiments.

### 5.2. Envenomation Modeling

The *Physalia* cnidome consists of several cnidae types, including two nematocysts: large and small heterotrichous anisorhizas [10,40]. Given their size and the length of their tubules (up to 1 mm long), the most important nematocysts for venom delivery are the bigger anisorhizas; we thus sought to quantify the discharge of these large, penetrant cnidae during a sting event. We utilized live human RBC from normal donors (approved protocol CHS#12561, University of Hawaii Committee on Human Studies) and low melting point agarose (Nusieve GTG Agarose, Lonza, Rockland, ME, USA) to constitute a live red blood cell agarose, a proxy for human tissue described in [14]. To calculate tentacle cnidae and cnidae discharge, approximately 1 cm of freshly cut tentacle was weighed, placed on a cover slip, and imaged for total length and width as well as bead length and width at 0.7× magnification (Olympus model SZX16, Olympus Corporation, Tokyo, Japan). Tentacle cnidae before stinging were imaged from below using an inverted scope at 10× magnification (Olympus model CKX41SF, Olympus Corporation, Tokyo, Japan). Tentacles were then placed on blood agarose and allowed to sting spontaneously for 10 min, with images for stinging length taken at 5 min into the sting duration. Images for length and width measurements as well as cnidae density were taken again post-sting. Adherent cnidae were photographed at 11.5× magnification (Olympus model SZX16, Olympus Corporation, Tokyo, Japan). All tentacle measurements and cnidae counts for density and discharge were determined from microscope images using ImageJ (U.S. National Institutes of Health, Bethesda, MD, USA).

### 5.3. Tentacle Solution Assay (TSA)

Method 1: Test for induction of discharge: Freshly cut tentacles (length 1–2 cm) were placed on clean, dry microscope slides and examined quickly for discharge; any lengths with notable discharge were discarded. 60 µL of the test solution was then added to the tentacle. After one minute of incubation, a cover slip was gently placed over the tentacle. Preliminary tests with seawater confirmed that coverslip addition did not induce significant discharge.

Method 2: Test for inhibition of chemically induced discharge: tentacles were treated as Method 1, but after 1 min of incubation, the test solution was carefully removed, and 60 μL of 100% ethanol was added. A coverslip was carefully placed after one minute of incubation with ethanol.

Method 3: Test for inhibition of pressure-induced discharge: tentacles were treated as Method 1, but after the 1-minute incubation and addition of the cover slip, the slide-tentacle-slip sandwich was pressed upon by hand for 30 s.

All photos were taken of the tentacles ten minutes after the various treatments through the coverslip using a dissecting microscope at 10× magnification (Olympus model CKX41SF, Olympus Corporation, Tokyo, Japan). Counts of discharged and undischarged cnidae were performed in ImageJ, and statistical analyses were performed using Graphpad Prism ver. 6.0 (GraphPad Software, La Jolla, CA, USA).

### 5.4. Tentacle Blood Agarose Assay

Effect of solutions and temperature treatments on sting severity was evaluated using a variation of the Tentacle Blood Agarose Assay (TBAA) ex vivo envenomation model outlined in [14]. Briefly, for *P. utriculus* studies, red blood cells were collected from normal human donors (approved protocol CHS#12561, University of Hawaii Committee on Human Studies) and suspended in a low melting point agarose to create an envenomation model where the area of hemolysis serves as a proxy for venom load. The agarose was comprised of 1% RBC and 1.5% Nusieve GTG Agarose (Lonza, Rockland, ME, USA) in modified RPMI (“YRPMI”: 23.81 mM NaHCO_3_, Fisher; 102.67 mM NaCl, BDH; 5.37 mM KCl, Fisher; 0.41 mM MgSO_4_·7H_2_O, Fisher; 25 mM HEPES, Fisher; 6.67 mM NaH_2_PO_4_, Fisher; 0.42 mM Ca(NO_3_)_2_·4H_2_O, Fisher). For *P. physalis* studies, premade sheep’s blood agar plates were used instead (Remel™, Lenexa, KS, USA). The two different media types used reflect the availability of appropriate laboratory space for work with human blood products.

For *P. utriculus*, blood agarose lengths (75 mm × 25 mm × 2 mm) were placed on glass slides and stung with whole fishing tentacles (~70 mm length) from freshly caught specimens. A 5-slide weight was then added to increase stinging. For rinse solution tests, the weight was removed after 5 min of stinging and 200 µL of the test solution was added to the tentacle; the weight was then replaced and the tentacle was allowed to sting for another 5 min. For temperature tests, the tentacles were allowed to sting for 10 min, with the weight briefly lifted at the 5-minute mark to mimic the rinse solution variant. After a total of 10 min of stinging, the weights and tentacles were removed, and saran-wrapped temperature packs were placed carefully over the slides and a mercury thermometer (Hot packs: Heat Wave XT reusable hand warmer, Bent Grass Concepts LLC, Greenwood Village, CO, USA; Cold packs: CVS Instant Cold Pack, CVS Pharmacy Inc., Woonsocket, RI, USA). For the room temperature replicates, a non-initiated hot pack was placed on the slides. Temperatures under the packs were recorded at 1, 2, 3, 4, 5, 10, 15 and 20 min. All slides were incubated at room temperature overnight, with photos taken at 5 and 24 h after the sting.

For *P. physalis*, standard 5% sheep’s blood agar plates were stung with 2- to 3-inch sections of fishing tentacles from freshly caught specimens. A glass slide was added as weight to increase stinging. For pretreament tests, tentacles were first incubated in test solutions for 2 min and rinsed twice for 2 min in seawater prior to 5 min of stinging. For temperature tests, the tentacles were allowed to sting for 5 min. After the tentacles were removed, and hot water packs (~45 °C) or ice packs were placed carefully over the plates. All plates were incubated at room temperature overnight, with photos taken at 3, 18 and 24 h after the sting.

Images were recorded using a dissecting microscope (Olympus model SZX16, Olympus Corporation, Tokyo, Japan) or a digital SLR camera (Fujifilm FinePix S4800, Fujifilm, Tokyo, Japan). The area of the zone of hemolysis was calculated using ImageJ. Briefly, scale was set using the known slide width and subsections (50 mm × 20 mm or 50 mm × 15 mm) were taken from each replicate for analysis to remove edge effects. Controls were used to set the color threshold for no hemolysis. The total area of the hemolytic zone was taken directly from the “analyze particles” function. Hemolytic zone was evaluated as the area exhibiting >80% hemolysis. Outliers were defined using the median absolute deviation (MAD) method detailed in [41], with the level of decision set conservatively at 3; any replicates which were outliers at all time points were removed. Shapiro–Wilk normality tests were conducted on all data sets from the 24-hour timepoint; if the data from 1/3 or more of the treatments was not normally distributed, then Kruskal–Wallis tests were used to compare means. Otherwise, one-way ANOVAs were used. All statistical analyses and post-hoc multiple comparisons were conducted in GraphPad Prism vers. 6.0 (GraphPad Software, Inc., La Jolla, CA, USA).

## Figures and Tables

**Table 1:** Comparison of the discharge of tentacle cnidae from two *Physalia* species in the TSA.

First-Aid Solution	Cnidae Discharge (%)	
	*P. physalis* (Atlantic)	*P. utriculus* (Pacific)
Seawater	00.59 ± 00.26	00.38 ± 00.13
Freshwater	40.94 ± 02.88 *	02.01 ± 00.61
Urine	42.54 ± 06.88 *	12.87 ± 02.60 *
Sting No More^®^ Spray	00.00 ± 00.00	00.06 ± 00.06
Pressure	46.64 ± 02.97 *	44.06 ± 13.79 *
Alcohols		
10% Ethanol	not tested	03.23 ± 01.32
30% Ethanol	not tested	03.90 ± 01.62
70% Ethanol	15.71 ± 03.68 *	13.58 ± 05.63 *
>95% Ethanol	33.96 ± 04.35 *	37.74 ± 02.78 *
Vinegars		
White Vinegar	01.04 ± 00.04	00.20 ± 00.20
Cider Vinegar	00.31 ± 00.13	00.69 ± 00.39
Malt Vinegar	00.47 ± 00.38	00.73 ± 00.45

Tentacles incubated in solutions for 10 min; pressure was applied for 30 s, with discharge read after 10 min. Numbers represent mean percent discharge ± standard error; sample sizes (*N*) were between 3 and 6. * Degree of discharge significantly greater than seawater (comparison of solutions only: one-way ANOVA with Fisher’s LSD post-hoc tests, *p* < 0.05). Overall, the two species reacted similarly to potential solutions, and strongly discharged in the presence of concentrated alcohols as well as in response to the application of pressure.

**Table 2:** Inhibition of cnidae discharge from *P. utriculus* tentacles after one-minute pretreatment with rinse solutions.

Test Solution	pH	Cnidae Discharge (%)	Ethanol-Induced Discharge (%)	Pressure-Stimulated Discharge (%)	Discharge Increase from Pressure (%)
Readily-Available					
Seawater	7.9	00.38 ± 0.13	37.74 ± 2.78	44.06 ± 13.79	*43.7*
Tap Water	6.0	02.01 ± 0.61	10.78 ± 6.85	15.50 ± 1.68 ^†^	*13.5*
Urine	6.2	12.87 ± 2.60 *	*—*	14.73 ± 3.91 ^†^	1.9
Shaving Cream	6.5	09.43 ± 6.45 *	*—*	22.45 ± 6.09 ^†^	*13.0*
Baking Soda (3:1 in Seawater)	9.0	08.90 ± 2.67 *	*—*	35.56 ± 7.24	*26.7*
Dish Soap	n/a	01.68 ± 0.30	09.40 ± 4.81	23.75 ± 3.34	*22.1*
Acetic Acid Solutions					
White Vinegar (3–5%)	2.5	00.20 ± 0.20	00.00 ± 0.00	02.78 ± 0.88 ^†^	2.5
Cider Vinegar (3–5%)	3.2	00.69 ± 0.39	00.00 ± 0.00	02.27 ± 0.29 ^†^	1.6
Malt Vinegar (3–5%)	2.9	00.73 ± 0.45	00.24 ± 0.20	04.46 ± 2.22 ^†^	3.7
Balsamic Vinegar (3–5%)	3.0	01.94 ± 1.13	00.80 ± 0.28	03.47 ± 1.22 ^†^	1.5
5% Acetic Acid in Distilled Water	2.4	00.50 ± 0.44	00.96 ± 0.42	02.61 ± 1.45 ^†^	2.1
75% White Vinegar in Seawater (~3.75%)	2.6	01.82 ± 0.49	01.50 ± 0.79	20.40 ± 5.79 ^†^	*18.6*
50% White Vinegar in Seawater (~2.5%)	2.6	01.11 ± 0.36	01.48 ± 0.50	24.34 ± 7.30	*23.2*
25% White Vinegar in Seawater (~1.88%)	2.6	00.87 ± 0.51	06.79 ± 3.50	26.05 ± 5.29	*25.2*
10% White Vinegar in Seawater (~0.5%)	2.7	01.66 ± 1.22	03.17 ± 1.70	31.35 ± 8.31	*29.7*
Other Acidic Solutions					
110 mM saline + HCl	2.0	15.90 ± 7.70 *	*—*	13.30 ± 1.48 ^†^	−2.6
Lemon Juice	4.0	12.40 ± 2.32 *	*—*	12.27 ± 3.09 ^†^	−0.1
Regular Cola	4.0	02.80 ± 1.91	07.72 ± 5.37	18.12 ± 11.07 ^†^	*15.3*
Alcohols					
Ethanol (>95%)	n/a	37.74 ± 2.78 *	*—*	41.06 ± 7.57	3.3
Isopropanol (>95%)	n/a	21.48 ± 6.76 *	*—*	24.25 ± 8.91	2.8
Commercial Products and Active Ingredients					
Sting No More^®^ Spray	3.0	00.00 ± 0.00	00.00 ± 0.00	03.53 ± 1.27 ^†^	3.5
Copper Gluconate (30 mM in 110 mM saline)	4.0	00.06 ± 0.06	05.11 ± 3.08	11.25 ± 1.49 ^†^	*11.2*
Magnesium Sulfate	4.9	08.57 ± 3.35 *	*—*	*—*	*—*
Lidocaine (4%)	4.6	00.50 ± 0.14	00.46 ± 0.31	51.22 ± 12.16	*50.7*

* Degree of discharge seen in solution-only test significantly greater than seawater (comparison of solutions only: one-way ANOVA with Fisher’s LSD post-hoc tests, *p* < 0.05), therefore inhibition tests were not necessarily performed. For each solution, the mean percent discharge ± standard error, are listed; sample sizes (*N*) were between 3 and 6. Significant differences in test solution direct effect was observed (one-way ANOVA, *p* < 0.001). Significant increases in discharge (post-hoc Sidak *p* < 0.05) are noted with an asterisk. Significant differences between solutions in response to pressure were also found (two-way ANOVA, *p* < 0.001). Significant reductions in pressure-induced discharge from seawater pressure-stimulated (post-hoc Sidak *p* < 0.05) are noted with ^†^. Discharge increase from pressure calculated as the solution only discharge subtracted from pressure stimulated discharge; increases in discharge with the application of pressure of more than ten percent (indicated with italics) represent particularly dangerous options if any form of scraping or rubbing is used to remove the tentacles and cnidae.

## Abbreviations

The following abbreviations are used in this manuscript:

TSBAA	Tentacle Skin Blood Agarose Assay
TBAA	Tentacle Blood Agarose Assay
TSA	Tentacle Solution Assay
Fisher’s LSD	Fisher’s Least Significant Difference

## Appendix A

**Table A1:** Inhibition of cnidae discharge from *P. physalis* tentacles (Florida) after one minute pretreatment with possible rinse solutions. Numbers represent mean percent discharge ± standard error, *N* = 3. All vinegars tested inhibited both chemically and mechanically stimulated cnidae discharge.

Test Solution	Cnidae Discharge (%)	Ethanol-Induced Discharge (%)	Pressure-Stimulated Discharge (%)
Seawater	00.59 ± 00.26	43.40 ± 02.17	46.64 ± 02.97
White Vinegar	01.04 ± 00.04	00.09 ± 00.09	00.81 ± 00.37
Cider Vinegar	00.31 ± 00.13	00.21 ± 00.17	00.65 ± 00.23
Malt Vinegar	00.47 ± 00.38	00.77 ± 00.47	not tested
Sting No More^®^ Spray	00.06 ± 00.06	00.03 ± 00.03	00.20 ± 00.20

**Table A2:** Cnidae discharge from *P. utriculus* tentacles with possible rinse solutions; Numbers represent mean percent discharge ± standard error. Difference between solution mean and seawater mean is reported with 95% confidence intervals (CI). Degree of discharge seen in solution-only test significantly greater than seawater (comparison of solutions only: one-way ANOVA); Fisher’s LSD post-hoc *p* values (compared to seawater) are reported (*p* < 0.05 in bold). Significant differences between solutions in response to pressure were also found (two-way ANOVA, *p* < 0.001); Difference between solution mean and seawater ^†^ pressure mean is reported with 95% confidence intervals as well as Sidak post-hoc *p* values (compared to seawater ^†^ pressure; *p* < 0.05 in bold).

Test Solution	Cnidae Discharge (%)	Mean Difference (95% CI)	*p* Value	Pressure-Stimulated Discharge (%)	Mean Difference (95% CI)	*p* Value
Seawater	00.38 ± 0.13			44.06 ± 13.79		
Tap Water	02.01 ± 0.61	−1.72 (−10.03–6.59)	0.6616	15.50 ± 1.68	33.28 (11.99–54.58)	**<0.0001**
Urine	12.87 ± 2.60	−12.58 (−20.8–4.27)	**0.002**	14.73 ± 3.91	29.33 (8.037–50.63)	**0.0008**
Shaving Cream	09.43 ± 6.45	−6.76 (−13.79–0.26)	**0.045**	22.45 ± 6.09	21.62 (0.32–42.91)	**0.0433**
Baking Soda (3:1 in Seawater)	08.90 ± 2.67	−8.61 (−16.92–0.3)	**0.0315**	35.56 ± 7.24	8.507 (−12.79–29.8)	0.9952
Dish Soap	01.68 ± 0.30	−1.39 (−9.7–6.92)	0.724	23.75 ± 3.34	20.31 (−0.99–41.6)	0.0768
White Vinegar (3–5%)	00.20 ± 0.20	0.09 (−8.22–8.4)	0.9825	02.78 ± 0.88	41.29 (19.99–62.58)	**<0.0001**
Cider Vinegar (3–5%)	00.69 ± 0.39	−0.4 (−8.71–7.91)	0.9184	02.27 ± 0.29	41.79 (20.49–63.09)	**<0.0001**
Malt Vinegar (3–5%)	00.73 ± 0.45	−0.45 (−8.76–7.86)	0.9095	04.46 ± 2.22	39.6 (18.31–60.9)	**<0.0001**
Balsamic Vinegar (3–5%)	01.94 ± 1.13	−1.65 (−9.96–6.66)	0.675	03.47 ± 1.22	40.6 (19.3–61.89)	**<0.0001**
5% Acetic Acid in Distilled Water	00.50 ± 0.44	−0.21 (−8.52–8.1)	0.9579	02.61 ± 1.45	41.45 (20.15–62.75)	**<0.0001**
75% White Vinegar in Seawater (~3.75%)	01.82 ± 0.49	−1.53 (−9.35–6.29)	0.6975	20.40 ± 5.79	23.66 (2.363–44.96)	0.0167
50% White Vinegar in Seawater (~2.5%)	01.11 ± 0.36	−0.83 (−8.65–6.99)	0.8337	24.34 ± 7.30	19.73 (−1.57–41.02)	0.0979
25% White Vinegar in Seawater (~1.88%)	00.87 ± 0.51	−0.59 (−8.41–7.23)	0.8809	26.05 ± 5.29	18.01 (−3.287–39.31)	0.1908
10% White Vinegar in Seawater (~0.5%)	01.66 ± 1.22	−1.37 (−9.19–6.45)	0.7278	31.35 ± 8.31	12.71 (−8.587–34.01)	0.7739
110 mM saline + HCl	15.90 ± 7.70	−15.61 (−23.92–7.3)	**0.0002**	13.30 ± 1.48	30.77 (9.47–52.06)	**0.0004**
Lemon Juice	12.40 ± 2.32	−12.12 (−20.42–3.81)	**0.0029**	12.27 ± 3.09	31.8 (10.5–53.1)	**0.0002**
Regular Cola	02.80 ± 1.91	−2.52 (−10.82–5.79)	0.523	18.12 ± 11.07	25.94 (4.647–47.24)	**0.0053**
Ethanol (>95%)	37.74 ± 2.78	−33.68 (−40.97–26.38)	**<0.0001**	41.06 ± 7.57	3.006 (−16.92–22.93)	>0.9999
Isopropanol (>95%)	21.48 ± 6.76	−21.2 (−29.5–12.89)	**<0.0001**	24.25 ± 8.91	19.82 (−1.48–41.11)	0.0943
Sting No More^®^ Spray	00.00 ± 0.00	0.29 (−8.02–8.6)	0.9417	03.53 ± 1.2 ^†^	41.39 (22.95–59.84)	**<0.0001**
30 mM Copper Gluconate	00.06 ± 0.06	0.23 (−8.08–8.54)	0.9532	11.25 ± 1.49	32.81 (11.52–54.11)	**0.0001**
Magnesium Sulfate	08.57 ± 3.35	−8.28 (−16.1–0.46)	**0.0383**	*—*	*—*	*—*
Lidocaine (4%)	00.50 ± 0.14	−0.21 (−7.9–7.49)	0.9545	51.22 ± 12.16	−7.153 (−28.45–14.14)	0.9996
